# Low back pain prevention behaviors and beliefs among the Polish population in a cross-sectional survey

**DOI:** 10.3389/fpubh.2024.1396558

**Published:** 2024-05-30

**Authors:** Paulina Kuśmierek, Mateusz Mikołajczyk, Dagmara Złotkowska, Anna Łowczak, Anita Mikołajczyk

**Affiliations:** ^1^Department of Human Physiology and Pathophysiology, University of Warmia and Mazury in Olsztyn, Olsztyn, Poland; ^2^Faculty of Medicine and Dentistry, Medical University of Warsaw, Warsaw, Poland; ^3^Department of Food Immunology and Microbiology, Polish Academy of Sciences, Olsztyn, Poland; ^4^Department of Pulmonology, University of Warmia and Mazury in Olsztyn, Olsztyn, Poland; ^5^Department of Psychology and Sociology of Health and Public Health, University of Warmia and Mazury in Olsztyn, Olsztyn, Poland

**Keywords:** low back pain, LBP, back pain prevention, work, cross-sectional survey

## Abstract

**Background:**

Low back pain (LBP) is one of the most common problems of public health and creates a burden globally. The aim was to assess the Polish population’s back pain prevention behaviors and beliefs and to examine how these health behaviors and beliefs vary across sociodemographic factors and physical activity.

**Methods:**

A cross-sectional survey was carried out among 208 randomly selected patients of the public general practitioner clinic. The differences in LBP-related beliefs and attitudes were determined due to participants’ status of requiring or non-requiring LBP treatment.

**Results:**

More than half of the respondents did not engage in behaviors that protect against back pain. Individuals with higher education levels and those who exercised at least once a week were significantly more likely to adopt behaviors to protect their backs. Less than half of the participants reported having a workplace that was adequately prepared to protect against back pain, and only 35.1% of the participants reported receiving instruction while taking up work on how to avoid back pain while working. According to respondents’ opinions, preventive actions are necessary to protect against back pain. Inappropriate exercises and stress can be contributors to back pain, with these opinions reported more often by women and participants with higher education levels. Participants who received treatment for LBP showed a significantly higher expression of behaviors to protect against back pain compared to participants who did not require treatment. However, there were no significant differences in participants’ beliefs about back pain prevention between the group requiring LBP treatment and the group not requiring LBP treatment.

**Conclusion:**

The study provides valuable insights into the association between LBP treatment, back pain prevention behaviors, and beliefs, suggesting potential avenues for future research and intervention development. By addressing workplace ergonomics and promoting a culture of back health, it may be possible to reduce the burden of LBP in Poland.

## 1 Introduction

Low back pain (LBP) is one of the most common problems of public health and creates a burden globally (1). In 2020, low back pain affected 619 million people globally, and it is projected more than 800 million people will have low back pain in 2050 (2). Low back pain is one of the main global causes of disability-adjusted life years (DALYs) (2). Disability-related low back pain has been at the highest level in working-age groups for years (3, 4). About 70–80% of people experience low back pain at least once in their life, especially in developed countries (5, 6). LBP is a very frequent symptom in all age groups, even in very young people (7, 8). Because back pain occurs among young people of reproductive age, this could have an impact on the economic situation both for them individually and for the government (7, 9).

In addition to being a common disorder worldwide, low back pain has a significant impact on quality of life and is associated with depressive symptoms (10, 11). Moreover, low back pain is one of the most common causes of sick leave and work absence. Wynne-Jones et al. (12) determined that 93% of people with back pain returned to work after 6 months or more. Saes-Silva et al. (11) showed that 31% of Brazilian adults with chronic back pain missed work. Incorrect sitting position could cause numerous musculoskeletal pain syndromes, mostly among people who spend many hours working with a computer (13). Work-related musculoskeletal symptoms, especially neck and low back pain, have been studied worldwide for many years, and their risk factors have been reported from various perspectives. Modifications at the workplace, such as re-organization of work, time work, improvement of work environment, postures during work and usability of machines, could shorten the time of returning to work, limit sickness absence and reduce back pain (14–16). It is necessary to draw attention to education on the principles of ergonomic sitting. The education of back pain prevention behaviors and beliefs from an early age of children can be crucial in back pain prevention. Adults who demonstrate healthy behaviors and correct beliefs about back pain prevention are positive role models for children. All preventive back pain interventions could play a significant role in improving quality of life and decreasing disability.

The aim of the study was to assess the Polish population’s back pain prevention behaviors and beliefs and to identify a specific demographic group that does not adhere to back pain prevention. A secondary aim was to examine how these health behaviors and beliefs vary across sociodemographic factors (including gender, education, age, and BMI) and physical activity longer than 30 min at least once a week. The differences in LBP-related beliefs and attitudes were determined due to participants’ status of requiring or non-requiring LBP treatment.

## 2 Materials and methods

### 2.1 Definitions and clarifications

LBP (low back pain) is defined as pain localized between the 12th ribs and the gluteal folds, with or without leg pain (17). Treatment for LBP depends on the nature of the pain and whether it is non-specific or specific. Pharmacological LBP management is the management of LBP with medications. Pharmacological and/or surgical treatments for LBP are connected with using medication such as for example, non-steroidal anti-inflammatory drugs, skeletal muscular drugs, opioids, or a combination of opioid and nonopioid analgesia. Non-pharmacological interventions for LBP means non-drug and non-invasive treatment in the management of LBP and include, for example, acupuncture, dry needling, education, electrophysical modalities, exercise programs, heat and cold therapies, manual therapies and psychological therapies (18–21).

Although nonpharmacologic treatment is preferred over pharmacologic treatment for the first-line management of LBP, they are commonly used together in clinical practice by doctors. Treatments for LBP can be provided by GPs or specialists such as rheumatologists, orthopedic spinal surgeons, pain management physicians, or other healthcare professionals, for example, physical therapists or osteopaths.

### 2.2 Participants, the inclusion and exclusion criteria

This study was a cross-sectional survey. The study was carried out among 208 randomly selected patients of the public general practitioner clinic between May 2022 and July 2023 in Poland. The inclusion criteria were as follows: age ≥ 18 years old. The exclusion criteria were individuals who had never experienced LBP and people who experienced LBP and undertook self-treatment without visiting a GP or other healthcare providers because of LBP.

Participants were divided into two groups. The first group (group requiring LBP treatment) included participants who had been pharmacologically or non-pharmacologically treated for LBP by GPs, specialists, or other healthcare providers. The second group (not requiring LBP treatment) included participants who did not require treatments for LBP. They had never experienced LBP that required pharmacological or non-pharmacological interventions.

### 2.3 Questionnaire

The questionnaire used for this study was created based on general principles (22). The questionnaire consisted of 11 main closed-ended items (related to behaviors to protect back pain, opinions about prevention of LBP and workplace adjustment to prevent back pain) and was supplemented with five demographic questions. The Polish and English questionnaires are presented in the Supplementary material. The verification of a proper understanding of the questionnaire’s items was conducted on a group of 20 pilot study participants. The questions were revised and modified.

The validation procedure of the questionnaire was conducted on a group of 50 respondents. The comprehensibility and acceptability of the questionnaire were independently evaluated by two public health specialists using a designed semi-structured interview. Most respondents indicated that the form of the questionnaire was good (98%) and sufficiently long (98%). All respondents claimed that the font size was big enough. For 48 respondents (96%) the questions were understandable. Only one individual indicated that there were questions he/she did not want to answer and only 12% of respondents said they found it difficult to answer some questions. The time taken for completion of the survey ranged from 1 to 15 min (median time 2 min). One-fifth of respondents (10 individuals) claimed that by completing this questionnaire they noticed important aspects related to LBP prevention that they had not recognized before participation in the current study. A reliability analysis of the questionnaire was used to estimate the compliance of the answers. The same questionnaire was done twice by the same group of participants in a two-week interval, and the degree of repeatability of responses was estimated by Kappa-Cohen’s coefficient. The values of the Kappa-Cohen’s coefficient range from 0 to 1, and values were interpreted as: 0.81–1.00 very good repeatability, 0.61–0.80 good repeatability, 0.41–0.60 moderate repeatability, 0.20–0.40 poor repeatability, <0.21 very poor repeatability (23). The results of the reliability analysis showed very good repeatability for all main items (Kappa-Cohen’s coefficient ranged from 0.92 to 1.00).

The self-administered questionnaire was distributed among participants by interviewers. No incentives were given to the participants. The interviewers recruited participants to study using selection questions. Respondents who were under the age of 18 and respondents who were involved in exclusion criteria (mentioned in section 2.2.) did not participate in the survey. The survey was voluntary and anonymous. Participants gave consent to participate by starting the survey.

The sample size for the current cross-sectional study was calculated to be representative based on previous studies (24–26) and our pilot study carried out on a group of 50 participants. In the pilot study, the Kish-Leslie formula (25) was used to determine the sample size sufficient for validating the findings. Based on the prevalence of LBP in analyzed group of 16%, a margin error of 5% and a standard normal deviation of (1.96) corresponding to the 95% confidence interval, the calculated sample included 206 participants.

### 2.4 Statistical analysis

Data were collected using a nominal scale (11 main items, gender, education) and a continuous scale (age, weight, height). The Body Mass Index (BMI) was calculated and used in the analysis as a continuous variable or a dichotomous variable (≤30 vs. >30). The subgroups of variable frequency of physical activity longer than 30 min were combined and used as a dichotomous variable (at least once a week vs. no exercise). Descriptive statistics were used to characterize the study group. The normality distribution of age and BMI was tested by the Shapiro–Wilk test. Non-parametric tests were used to estimate the differences in participants’ behaviors and beliefs for protecting back pain due to continuous variables (age and BMI) using the Mann–Whitney test (for two subgroups) or Kruskal-Wallis test (for >2 subgroups) and due to categorical variables (gender, education, physical activity status and participants’ status of requiring or non-requiring LBP treatment) using the chi-square test. A *p*-value <0.05 was considered to be significant. The analysis was conducted using Statistica (data analysis software system), version 13. https://www.statsoft.pl/statistica_13/ (accessed on 1 August 2022).

## 3 Results

### 3.1 Characteristics of participants

The study involved 208 respondents between the ages of 18 and 82 years, with a median age of 39 years. Among them, 85 were men (40.9%), and 123 were women (59.1%). Almost half of the participants had completed secondary school (47.6%). BMI ranged from 18 to 44, with a median BMI of 25. A total of 30 respondents (14.4%) had a BMI greater than 30, indicating obesity. Regarding physical activity, one-fifth of the respondents (20.2%) reported exercising for more than 30 min once a week, while only about 5% engaged in physical activity more than three times a week. Half of the participants did not exercise regularly. Furthermore, one-third of the respondents (37.5%) reported spending their free time with little or no physical activity. Almost half of the participants (49.5%) did not exercise regularly, and only one-fifth of the respondents performed physical exercise for more than 30 min once a week (Table 1).

**Table 1:** Characteristics of participants.

Age median (25–75% IQR)		39 (32–48)
BMI median (25–75% IQR)		25 (23–28)
Gender *n* (%)		
	Male	85 (40.9)
	Female	123 (59.1)
Education *n* (%)		
	Primary	17 (8.2)
	Secondary	99 (47.6)
	High	88 (42.3)
	No data	4 (1.9)
Frequency of physical activity longer than 30 min *n* (%)		
	Once a week	42 (20.2)
	Twice a week	31 (14.9)
	Three times a week	21 (10.1)
	More than three times a week	11 (5.3)
	No exercise	103 (49.5)

IQR – interquartile range.

### 3.2 Behaviors for preventing back pain

**Figure 1:**
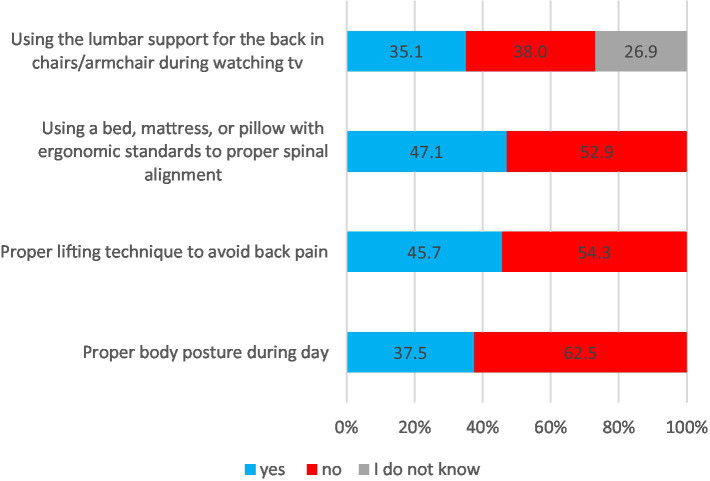
Behaviors to protect back pain.

More than half of the respondents did not exhibit behaviors that protect against low back pain. Specifically, only 35% of participants used lumbar support for their back while sitting in chairs or armchairs during TV watching. Additionally, 47% of respondents used a bed, mattress, or pillow with ergonomic standards to maintain proper spinal alignment. Furthermore, 45.7% of respondents demonstrated a proper lifting technique to avoid back pain, and 37.5% maintained a proper body posture throughout the day (Figure 1).

Women had better posture during the day than men, according to respondents (46% vs. 25%; *p* = 0.002), more graduated from high school than secondary school (55% vs. 25%, *p* < 0.001), were younger (median age 38 vs. 40; *p* = 0.02), with lower BMI (*p* < 0.001) and were physically active at least a once a week compared with those who did not exercise (44% vs. 31%, *p* = 0.06) (Table 2).

**Table 2:** Behaviors and beliefs for preventing back pain by demographic factors and physical activity.

		Gender					Education					Age			BMI				Physical activity longer than 30 min				
		Female *n* (%)		Male *n* (%)		*p*	Secondary *n* (%)		High *n* (%)		*p*	Median	(25–75% IQR)	*p*	Median	(25–75% IQR)		*p*	At least once a week *n* (%)		No exercise *n* (%)		*p*
Beliefs about back pain prevention	Inappropriate exercises could have a negative effect on the back																						
	Yes	91	(75)	53	(62)	0.10	60	(61)	72	(83)	<0.001	39	(32–48)	0.09	25	(22–27)		0.05	74	(71)	70	(68)	0.05
	No	8	(6)	5	(6)		5	(5)	7	(8)		38	(29–43)		23	(22–24)			10	(10)	3	(3)	
	I do not know	23	(19)	27	(32)		34	(34)	8	(9)		42	(35–53)		27	(23–30)			20	(19)	30	(29)	
	Back pain could be related to stress																						
	Yes	56	(46)	30	(35)	0.02	40	(40)	42	(48)	0.20	38	(32–45)	0.48	24	(22–27)		0.06	46	(44)	40	(39)	0.32
	No	27	(22)	11	(13)		15	(15)	18	(20)		40	(36–48)		24	(22–26)			15	(14)	23	(22)	
	I do not know	40	(32)	44	(52)		44	(45)	28	(32)		39	(31.5–52)		25	(23–29)			44	(42)	40	(39)	
	Back preventive actions need to be taken to protect back pain																						
	Yes	107	(87)	63	(74)	0.06	80	(81)	75	(85)	0.44	40	(33–50)	0.10	25	(23–28)		0.18	84	(80)	86	(83)	0.62
	No	3	(2)	4	(5)		4	(4)	1	(1)		41	(22–66)		22	(22–24)			3	(3)	4	(4)	
	I do not know	13	(11)	18	(21)		15	(15)	12	(14)		38	(26–44)		24	(22–27)			18	(17)	13	(13)	
Behaviors to protect back pain	Proper body posture during the day																						
	Yes	57	(46)	21	(25)	0.002	25	(25)	48	(55)	<0.001	38	(30–43)	0.02	24	(22–26)		<0.001	46	(44)	32	(31)	0.06
	No	66	(54)	64	(75)		74	(75)	40	(45)		40	(33–51)		25	(23–29)			59	(56)	71	(69)	
	Proper lifting technique to avoid back pain																						
	Yes	58	(47)	37	(44)	0.61	30	(30)	56	(64)	<0.001	39	(36–45)	0.97	25	(22–27)		0.19	59	(56)	36	(35)	0.002
	No	65	(53)	48	(56)		69	(70)	32	(36)		40	(31–51)		25	(23–29)			46	(44)	67	(65)	
	Using a bed, mattress, or pillow with ergonomic standards for proper spinal alignment																						
	Yes	61	(50)	37	(44)	0.39	37	(37)	50	(57)	0.008	40	(34–51)	0.09	25	(23–27)		0.37	62	(59)	36	(35)	<0.001
	No	62	(50)	48	(56)		62	(63)	38	(43)		39	(31–46)		25	(22–28)			43	(41)	67	(65)	
	Using the lumbar support for the back in chairs/armchairs while watching TV																						
	Yes	43	(35)	30	(35)	0.40	28	(28)	32	(36)	0.47	43	(36–55)	0.006	25	(23–28)		0.72	40	(38)	33	(32)	0.37
	No	43	(35)	36	(43)		41	(42)	34	(39)		37	(30–43)		24	(23–27)			35	(33)	44	(43)	
	I do not know	37	(30)	19	(22)		30	(30)	22	(25)		40	(33–47)		25	(22–28)			30	(29)	26	(25)	

Respondents who had a proper lifting technique to avoid back pain significantly more frequently graduated from high school compared with secondary school (64% vs. 30%; *p* < 0.001) and were physically active at least once a week in comparison with those who did not exercise (56% vs. 35%; *p* = 0.002) (Table 2).

Respondents who graduated high school used a bed, mattress, or pillow with ergonomic standards for proper spinal alignment more often than participants after secondary education (57% vs. 37%; *p* = 0.008). Similarly, once a week, physically active respondents more often used a bed, mattress, or pillow with ergonomic standards for proper spinal alignment in comparison with those who did not exercise (59% vs. 35%; *p* < 0.001) (Table 2). Older respondents more often used the lumbar support for the back in chairs/armchairs while watching TV than younger respondents (median age 43 vs. 37; *p* = 0.006) (Table 2).

### 3.3 Workplace

**Figure 2:**
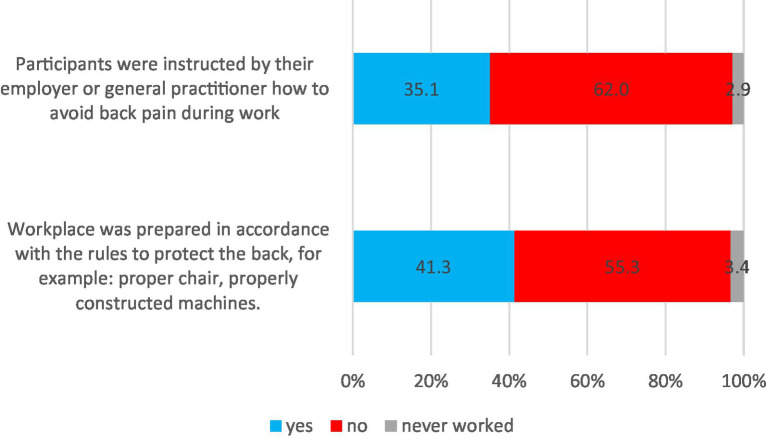
Back pain protection and the workplace.

More than half of the participants (55.3%) indicated that their workplace was not prepared in accordance with the rules to protect the back, such as proper chairs or adequately constructed machines. Only 35.1% of participants were instructed by their employer or general practitioner on how to avoid back pain during work (Figure 2).

Participants who were instructed by their employer or general practitioner on how to avoid back pain during work significantly more often claimed that their workplace was prepared in accordance with the rules to protect the back (respectively: 67% vs. 33%; *p* < 0.001).

### 3.4 Beliefs about back pain prevention

**Figure 3:**
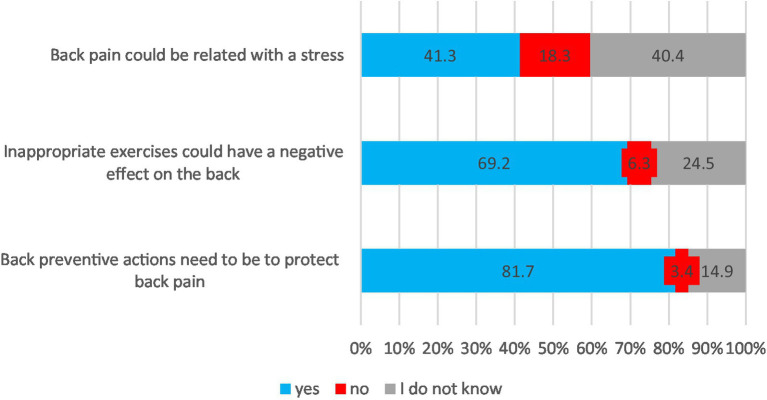
Beliefs about back pain prevention.

In general, respondents believed that taking preventive actions is necessary to protect against back pain (81.7%), and that inappropriate exercises could have a negative effect on the back (69.2%). Additionally, they believed that back pain could be related to stress (41.3) (Figure 3).

Women significantly more often thought that back pain could be related to stress than men (46% vs. 35%; *p* = 0.02). Respondents who graduated from high school more often reported negative back effects from inappropriate exercises compared to secondary school participants (83% vs. 61%; *p* < 0.001) (Table 2).

### 3.5 Associations between participants’ status of requiring or non-requiring LBP treatment and demographic factors, physical activity, behaviors, and beliefs for preventing back pain

A total of 96 participants (46%) experienced low back pain (LBP), and they had been pharmacological or non-pharmacologically treated for LBP by GPs, specialists or other healthcare providers (Group 1, requiring LBP treatment). In total, 112 participants (54%) did not require treatments for LBP (Group 2, non-requiring LBP treatment). Participants in Group 1 were significantly older than participants in Group 2 (median age 43 years vs. 37 years; *p* < 0.001). Participants who required LBP treatment had more frequent BMI > 30 than participants who had never been treated for LBP (22% vs. 8%; *p* = 0.005) (Table 3).

**Table 3:** Correlations between participants’ status of requiring or non-requiring LBP treatment and demographic factors.

		Group 1		Group 2		*p*
Age median (25–75% IQR)		43 (37–52)		37 (29–43)		<0.001
BMI *n* (%)						
	≤30	75	(78.1)	103	(92.0)	0.005
	>30	21	(21.9)	9	(8.0)	
Gender *n* (%)						
	Male	36	(37.5)	49	(43.8)	0.36
	Female	60	(62.5)	63	(56.2)	
Education *n* (%)						
	Secondary	46	(54.1)	53	(52.0)	0.77
	High	39	(45.9)	49	(48.0)	
Physical activity longer than 30 min *n* (%)						
	Minimum once a week	44	(45.8)	61	(54.5)	0.21
	No exercise	52	(54.2)	51	(45.5)	

Participants who were treated for LBP, in comparison with participants who did not require treatments for LBP, claimed that they significantly more often used a bed, mattress, or pillow with ergonomic standards for proper spinal alignment (58.3% vs. 37.5%; *p* = 0.003) and the lumbar support for the back in chairs/armchair while watching TV (44.8% vs. 26.8%; *p* = 0.03). There were no significant differences in participants’ beliefs about back pain prevention due to participants’ status of requiring or non-requiring LBP treatment (Table 4).

**Table 4:** Correlations between participants’ status of requiring or non-requiring LBP treatment and their behaviors and beliefs for protecting back pain.

		Group 1 *n* (%)		Group 2 *n* (%)		*p*
Beliefs about back pain prevention	Inappropriate exercises could have a negative effect on the back					0.46
	Yes	67	(69.8)	77	(69.4)	
	No	4	(4.2)	9	(8.1)	
	I do not know	25	(26.0)	25	(22.5)	
	Back pain could be related to stress					0.87
	Yes	39	(40.6)	47	(42.0)	
	No	19	(19.8)	19	(17.0)	
	I do not know	38	(39.6)	46	(41.0)	
	Back preventive actions need to be taken to protect against back pain					0.58
	Yes	80	(83.3)	90	(80.4)	
	No	4	(4.2)	3	(2.7)	
	I do not know	12	(12.5)	19	(16.9)	
Behaviors to protect back pain	Proper body posture during the day					0.08
	Yes	42	(43.8)	36	(32.1)	
	No	54	(56.2)	76	(67.9)	
	Proper lifting technique to avoid back pain					0.38
	Yes	47	(49.0)	48	(42.9)	
	No	49	(51.0)	64	(57.1)	
	Using a bed, mattress, or pillow with ergonomic standards for proper spinal alignment					0.003
	Yes	56	(58.3)	42	(37.5)	
	No	40	(41.7)	70	(62.5)	
	Using the lumbar support for the back in chairs/armchairs while watching TV					0.03
	Yes	43	(44.8)	30	(26.8)	
	No	31	(32.3)	48	(42.9)	
	I do not know	22	(22.9)	34	(30.3)	

## 4 Discussion

The findings of this cross-sectional survey provide valuable insights into the selected LBP prevention behaviors and beliefs among the Polish population and their associations with demographic factors and physical activity. Physical activity levels were relatively low, with only 20.2% of respondents exercising for longer than 30 min once a week. Previous research has shown that physical inactivity is a common risk factor for LBP (7, 27). Regarding behaviors related to LBP prevention, more than half of the respondents did not engage in preventive practices. For instance, only 35% of participants used lumbar support for the back in chairs during TV watching, and less than half (47%) used a bed, mattress, or pillow with ergonomic standards for proper spinal alignment. Furthermore, only 45.7% of respondents reported adopting a proper lifting technique to avoid back pain. These results suggest a need for greater awareness of preventive behaviors promotion, such as ergonomic standards for sitting, sleeping, and lifting heavy objects to minimize strain on the spine. Comfortable mattresses that meet ergonomic standards can improve sleep quality and alleviate back pain. Ancuelle et al. (28) showed a significant reduction of cervical dorsal and lumbar pain after 4 weeks of using a properly selected mattress. Other studies determined the relationship between LBP and inadequate posture and the way of lifting objects. Squat lifting often remains the recommended lifting technique, but the findings of von Arxet et al. (29) study provide evidence that there is no one-size-fits-all approach, especially when considering that squat lifting produced higher anterior shear forces in the L5/S1 segment. It should be pointed out that not only the kind of lifting technique is vital in avoiding spinal loads but also the duration of the lifting task (30) and awareness of using a lifting technique that optimizes movement by calming tissue down (31). The current results suggest a need for greater preventive awareness and promotion of preventive behaviors to reduce the burden of LBP in the Polish population. The results indicate that there is room for improvement in promoting back pain prevention practices, as more than half of the respondents did not engage in behaviors to protect against back pain (Figure 1). This highlights a significant gap in back pain prevention efforts within the Polish population and aligns with previous studies highlighting the global burden of LBP and its impact on disability-adjusted life years (DALYs) and healthy life expectancy (HALE) (3).

Interestingly, the current study identified certain demographic factors associated with back pain prevention behaviors. Women were more likely than men to maintain a proper body posture during the day. Additionally, participants with higher education levels and those who engaged in physical activity at least once a week demonstrated more frequent use of ergonomic beds, mattresses, or pillows. There were encouraging trends observed among individuals with higher education levels and those who engaged in physical activity for at least 30 min once a week (Table 2). These subgroups demonstrated significantly higher adherence to back pain prevention practices by a higher likelihood of adopting behaviors to protect their back, suggesting that educational attainment and regular physical activity could be potential factors promoting back health awareness and proactive measures. These findings are consistent with previous research emphasizing the relationship between education and physical activity with back pain prevention behaviors (32–34).

The current study also highlighted workplace-related issues concerning back pain prevention. Less than half of the participants reported having a workplace adequately prepared to protect the back, and more than half of the participants indicated that their workplace was not prepared in accordance with the rules to protect the back, such as proper chairs and adequately constructed machines. Another concerning finding was that only a minority of the participants received instructions from their employers or occupational physicians on how to avoid back pain during work (Figure 2). This lack of emphasis on workplace ergonomics and back health aligns with previous studies showing the importance of addressing work-related risk factors for LBP (12, 35). Improper working conditions and lack of awareness of ergonomic issues could be a reason for low back pain (36). A systematic review (13) proved that incorrect sitting posture and spending many hours in sedentary working conditions with minimal physical activity could be a reason for many musculoskeletal disorders. Work from home, and LBP may be associated with the quality of the work environment. In Japan, the association between the frequency of teleworking in poor work conditions at home and LBP was reported, especially during the COVID-19 pandemic (37, 38). Uncomfortable posture, insufficient room to concentrate, inadequate lighting, desk space and foot space, and cold temperature could be risk factors for LBP among workers who work from home (39, 40). Additionally, the lack of room to work at home could generate psychological stress, which seems to be also a risk factor for LBP (41–43). Identifying and diminishing work-related psychosocial stressors can be paramount in combating the high prevalence of LBP in the working environment (42). The workers, including health workers (26) need ergonomic job organization, courses on correct lifting techniques, and building health public policies for new worker recruits.

Absence from work due to back pain or musculoskeletal disorders can be a big problem for both individuals and companies. Some authors also determined that musculoskeletal pain was a risk for long-term work absence (33). Watanabe et al. (44) showed that 35.4% of shipyard workers experienced absence from work due to back pain. A systematic review and meta-analysis (12) also confirmed that most people with back pain return to work after at least 6 months or more. A potential lack of emphasis on workplace ergonomics and back health is a big problem. Addressing this issue could lead to significant improvements in preventing work-related lower back pain and reducing the associated economic burden and productivity losses.

In the current study, the participants’ beliefs about back pain prevention were also assessed, and it was found that respondents perceived back preventive actions as necessary efforts in protection against back pain. Most of the participants claimed that inappropriate exercises could have a negative effect on the back (Figure 3). Our findings align with previous studies that individuals have a predominant belief in the potentially back-damaging effects of activity exercise (45, 46). However, there was significant variation in beliefs regarding preventing back pain based on gender and educational status. Participants who graduated from secondary school, in comparison with high school participants, expressed such beliefs less often. Opinions that inappropriate exercises and stress can be a contributor to back pain were more frequently expressed by women and individuals with higher education (Table 2). This suggests that targeted educational interventions, particularly tailored to these demographic groups, may be effective in promoting a culture of back health awareness and preventive measures (32). Tan et al. (32) determined that younger Chinese healthcare professionals and those working in regional community health centers had more frequent negative LBP-related beliefs, including beliefs about the inevitable consequences of back pain. Other authors showed that back pain beliefs could differ due to culture, education, place of employment or LBP disability (35, 47).

The study also explored the differences between participants who had received treatment for LBP and those who had not. In the current study, participants who required LBP treatment tended to be older and have a higher body mass index (BMI > 30) compared to those who had never been treated (Table 3). This finding highlights the importance of addressing age and obesity as potential risk factors for LBP and implementing targeted preventive strategies in these high-risk groups. Similar results were obtained by Jonsdottir et al. (48), who demonstrated that increased BMI > 30 and older age was related to the prevalence of acute back pain. Other authors also confirmed that increasing age and obesity are associated with back pain (49–51). Moreover, obesity is a risk factor for LBP-related disability (52). Interestingly, participants who required LBP treatment demonstrated a higher frequency of expressing behaviors to protect against back pain compared to those who had never received treatment (Table 4). This could be attributed to increased awareness and understanding of back pain management and prevention behaviors among individuals who had experienced LBP first-hand. These findings align with previous studies that emphasize the importance of learning from previous LBP experiences to promote better preventive behaviors (14, 16). However, there were no significant differences in participants’ beliefs about back pain prevention between those treated and those untreated for LBP. This suggests that while treatment experiences may influence behavior, there might be a shared understanding of beliefs for protecting back pain across the study population.

Overall, this cross-sectional survey sheds light on the state of low back pain prevention behaviors and beliefs among the Polish population. Educational programs focused on promoting back pain prevention practices, particularly among individuals with lower educational attainment and sedentary lifestyles, could lead to a reduction in the burden of LBP in Poland (53). Moreover, addressing workplace ergonomics and promoting preventive actions could have a significant impact on reducing work-related LBP and improving overall well-being and productivity among the workforce (15, 36).

It is essential to acknowledge some limitations of this study, including the cross-sectional design, which limits causal inferences. In the current study, there were no collected data of characteristics of low back pain (kind of LBP, time of LBP), except information on whether patients with LBP require or non-require treatment. A standardized pain rating scale was not used to assess pain intensity. Longitudinal studies could provide further insights into the factors influencing the adoption of back pain prevention behaviors over time. Additionally, self-reported data may be subject to recall bias or social desirability bias. Future research could incorporate objective measures and employ a mixed-methods approach to gain a more comprehensive understanding of the factors influencing back pain prevention behaviors and beliefs.

Synthesis of key points to conclude all obtained and discussed results:

(1) More than half of the respondents did not engage in behaviors that protect against back pain. (2) Individuals with higher education levels and those who exercised at least once a week were significantly more likely to adopt behaviors to protect their backs. (3) Less than half of the participants reported having a workplace that was adequately prepared to protect against back pain, and only 35.1% of the participants reported receiving instruction while taking up work on how to avoid back pain while working. (4) According to respondents’ opinions, preventive actions are necessary to protect against back pain. Inappropriate exercises and stress can be contributors to back pain, with these opinions reported more often by women and participants with higher education levels. (5) Participants who required treatment for LBP were significantly older and had a BMI > 30 compared to participants who had never been treated for LBP. (6) Participants who received treatment for LBP showed a significantly higher expression of behaviors to protect against back pain compared to participants who did not require treatment. However, there were no significant differences in participants’ beliefs about back pain prevention between the group requiring LBP treatment and the group not requiring LBP treatment.

## 5 Conclusion

In closing, this study contributes valuable information on LBP prevention behaviors and beliefs among the Polish population. The findings underscore the importance of targeted interventions to promote health awareness and preventive practices, particularly among vulnerable demographic groups. By addressing workplace ergonomics and promoting a culture of back health, it may be possible to reduce the burden of LBP and improve the overall quality of life for individuals in Poland. Finally, the study provides valuable insights into the association between LBP treatment, back pain prevention behaviors, and beliefs, suggesting potential avenues for future research and intervention development.

## Data availability statement

The raw data supporting the conclusions of this article will be made available by the authors, without undue reservation.

## Ethics statement

Ethical approval was not required for the study involving humans in accordance with the local legislation and institutional requirements. Written informed consent to participate in this study was not required from the participants or the participants’ legal guardians/next of kin in accordance with the national legislation and the institutional requirements.

## Author contributions

PK: Conceptualization, Data curation, Formal analysis, Investigation, Methodology, Project administration, Visualization, Writing – original draft. MM: Conceptualization, Data curation, Formal analysis, Investigation, Methodology, Project administration, Visualization, Writing – original draft. DZ: Writing – original draft. AŁ: Writing – original draft. AM: Conceptualization, Data curation, Formal analysis, Investigation, Methodology, Project administration, Supervision, Visualization, Writing – review & editing.
