# Supplementary material for: Low back pain prevention behaviors and beliefs among the Polish population in a cross-sectional survey
Source: Front Public Health. 2024 May 30;12:1396558. doi: 10.3389/fpubh.2024.1396558 (PMC11169837; doi:10.3389/fpubh.2024.1396558)
Supplement: Supplementary file 1 [file Data_Sheet_1.pdf]

Questionnaire

1. Have you ever experienced low back pain requiring pharmacological and/or non-pharmacological treatment?

☐ Yes
☐ No

2. In your daily life, do you pay attention to maintaining proper body posture during the day?

☐ Yes
☐ No

3. When lifting heavy objects, do you try to do it in a way that minimizes strain on your spine?

☐ Yes
☐ No

4. When choosing a bed, mattress, or pillow, did you pay attention to their ergonomic standards of spine disorder prevention?

☐ Yes
☐ No

5. Do you use the lumbar support for the back in chairs/armchairs while watching TV?

☐ Yes
☐ No
☐ I do not know

6. How often do you have physical activity longer than 30 minutes?

☐ Once a week
☐ Twice a week
☐ Three times a week
☐ More than three times a week
☐ No exercise

7. Do you believe that inappropriate exercises could have a negative effect on the back?

☐ Yes
☐ No, exercises never harm the spine
☐ I do not know

8. Do you believe that back pain could be related to stress?

☐ Yes
☐ No

- I do not know

9. Have you ever been instructed by an employer or occupational medicine physician how to avoid overloading the spine during future work?

- Yes
- No
- I have never worked

10. Have you ever had your workplace prepared in accordance with the rules to protect the back, for example: proper chairs and properly constructed machines?

- Yes
- No
- I have never worked

11. Do you think preventive actions are needed to protect back pain?

- Yes
- No
- I do not know

Demographic data

1. Gender

- Male
- Female

2. Education

- High
- Secondary
- Primary

3. Age……..……. years

4. Weight………. Kg

5. Height ………. cm
