# Supplementary material for: Low back pain prevention behaviors and beliefs among the Polish population in a cross-sectional survey
Source: Front Public Health. 2024 May 30;12:1396558. doi: 10.3389/fpubh.2024.1396558 (PMC11169837; doi:10.3389/fpubh.2024.1396558)
Supplement: Supplementary file 2 [file Data_Sheet_2.pdf]

ANKIETA

1. Czy kiedykolwiek wystąpiły u Pana(i) bóle krzyża wymagające farmakologicznego i/lub niefarmakologicznego leczenia?
- [ ] tak
- [ ] nie

2. Czy w życiu codziennym zwraca Pan(i) uwagę na utrzymywanie prawidłowej postawy ciała w ciągu dnia?
- [ ] tak
- [ ] nie

3. Czy podnosząc cięższe przedmioty, zwraca Pan(i) uwagę, aby wykonać tę czynność w sposób jak najmniej obciążający kręgosłup?
- [ ] tak
- [ ] nie

4. Czy wybierając łóżko, materac, czy poduszkę do spania, zwracał/a Pan(i) uwagę na kryteria, jakim powinien odpowiadać tego typu sprzęt, aby spełniać wymagania profilaktyki schorzeń kręgosłupa?
- [ ] tak
- [ ] nie

5. Czy oglądając telewizję, korzysta Pan(i) z krzesła lub fotela, zapewniającego podparcie lędźwiowego odcinka kręgosłupa?
- [ ] tak
- [ ] nie
- [ ] nie wiem

6. Ile razy w tygodniu wykonuje Pan(i) ćwiczenia fizyczne trwające powyżej 30 minut?
- [ ] jeden raz w tygodniu
- [ ] dwa razy w tygodniu
- [ ] trzy razy w tygodniu
- [ ] częściej niż trzy razy w tygodniu
- [ ] nie ćwiczę

7. Czy uważa Pan(i), że nieodpowiednio dobrany zestaw ćwiczeń, może być szkodliwy dla kręgosłupa?
- [ ] tak
- [ ] nie, ćwiczenia nigdy kręgosłupowi nie szkodzą
- [ ] nie wiem

8. Czy uważa Pan(i), że dolegliwości bólowe kręgosłupa mogą mieć związek ze stresem?
- [ ] tak

- [ ] nie
- [ ] nie mam zdania w tej kwestii

9. Czy przy podejmowaniu zatrudnienia kiedykolwiek był(a) Pan(i) poinstruowany(a) (np.: przez pracodawcę lub lekarza medycyny pracy) o tym, w jaki sposób należy unikać przeciążeń kręgosłupa podczas wykonania przyszłej pracy?

- [ ] tak
- [ ] nie
- [ ] nigdy nie pracowałem/am zawodowo

10. Czy uważa Pan(i), że którekolwiek stanowisko Pana(i) pracy jest lub było urządzone zgodnie z zasadami uwzględniającymi dbałość i ochronę kręgosłupa np.: odpowiednie krzesło, właściwie skonstruowane maszyny?

- [ ] tak
- [ ] nie
- [ ] nigdy nie pracowałem/am zawodowo

11. Czy Pana(i) zdaniem należy podejmować działania profilaktyczne, aby zapobiegać bólom kręgosłupa?

- [ ] tak
- [ ] nie
- [ ] nie mam zdania na ten temat

Metryczka

1. Płeć

- [ ] mężczyzna
- [ ] kobieta

2. Wykształcenie

- [ ] wyższe
- [ ] średnie
- [ ] podstawowe

3. Wiek.............. lat

4. Waga …………. Kg

5. Wzrost ………. cm
